# Weak Associations of Morningness-Eveningness and Stability with Skin Temperature and Cortisol Levels

**DOI:** 10.5334/jcr.182

**Published:** 2019-07-16

**Authors:** Corina Weidenauer, Christian Vollmer, Katharina Scheiter, Christoph Randler

**Affiliations:** 1 University of Tübingen, DE

**Keywords:** Morningness-Eveningness, MESSi, skin temperature, cortisol awakening response

## Abstract

Differences in daytime preferences can be described on the dimension of morningness-eveningness (continuous) or circadian typology (categorical) and are associated with our physiological functioning, which is reflected in body temperature and cortisol levels in the morning. The purpose of the present study was to explore the relationship between morningness-eveningness, stability and physiological markers (body temperature and cortisol) based on a three-dimensional conceptualization of morningness-eveningness using the Morningness-Eveningness-Stability Scale improved (MESSi). In contrast to previously used unidimensional measures, the MESSi determines circadian typology and its amplitude in three dimensions: Morning affect (MA), Eveningness (EV) and Stability/Distinctness (DI). Furthermore, the differences of the cortisol levels between weekday and weekend were examined. The sample (*N* = 42) consisted of extreme chronotypes (age 18–54 years; *M* = 24.8 years, *SD* = 5.83; 22 morning types [5 men and 17 women] and 20 evening types [8 men and 12 women]). The participants were asked to measure their skin temperature for one week and sample four saliva probes for cortisol determination. Morning types showed a better fit in the actual temperature data to the approximating data as compared to Evening types and showed a higher overall temperature. The Stability/Distinctness (DI) component of the MESSi was negatively correlated with the nadir. Morning types also showed higher cortisol levels than Evening types immediately after awakening. The cortisol levels were higher on a weekday compared to the weekend. To conclude, the present findings demonstrate that the skin temperature is weakly associated with morningness-eveningness and the stability of the circadian phase.

## 1. Introduction

Human biological rhythms show continuous fluctuations in a nearly 24-hour range (circadian rhythm) [1]. The distribution of active and rest phases or sleep and wakefulness differs between individuals [2]. These individual daytime preferences can be summarized as morningness-eveningness (M/E). Research has identified three different groups of so-called “chronotypes”: morning types (M-types), neither types, and evening types (E-types). M-types (“larks”) prefer to wake up and go to bed early and tend to have their mental and physical peak in performance during the first half of the day. In contrast, E-types (“owls”) prefer to get up late and go to bed late. Their peak in performance can be found during the second half of the day [3]. It is well known that circadian typology affects many areas of daily life, like cognition [4], eating habits [5], and mental disorders [6]. Therefore, it is important to correctly assess and estimate the individual circadian phase. This can be done on the one hand by using certain physiological and biological markers such as: melatonin secretion [7], cortisol measurement [8], body temperature recording [9] or gene-expression analysis by blood and epidermis samples [10,11]. On the other hand, self-assessments can be used as a convenient way, especially when conducting research in large samples.

### 1.1 The MESSi

M/E can be measured by using different self-assessments. Depending on the purpose and context of the planed study, one can choose for example between the most commonly used Morningness-Eveningness Questionnaire (MEQ) by Horne and Östberg [12], the Diurnal Type Scale (DTS) by Torsvall and Åkerstedt [13] or the Munich ChronoType Questionnaire (MCTQ) by Roenneberg and his colleagues [14,15,3]. However, all of these measures refer to M/E as a unidimensional construct, with morningness and eveningness marking the two endpoints of one scale. For example, the MEQ only determines circadian preference on a specific phase of the day and categorizes the participants into groups by certain cut-offs [12]. However, M/E should be thought of and measured as a multidimensional construct, where morningness, eveningness and stability refer to different dimensions. The dimension morningness describes the affective facet of the morning orientation and takes for example into account how easy it is to get out of bed in the morning or how awake someone feels. The dimension eveningness determines the affective facet of the evening orientation and also evaluates activity aspects and mood in the evening. The third dimension distinctness/stability operationalizes the fact that circadian preferences fluctuates throughout the day. Subjective feelings, concentration and motivation plays also an important role [16]. Although M/E has been seen as an one-dimensional trait [15] some researches indicate that M/E should be determined by two separated dimensions [17]. Further it has been suggested, that the amplitude of the circadian preference should also been taken into account and should be an additional measure [18,19]. Thereby, the amplitude of the circadian preference describes the range of the fluctuations in circadian preference.

The Morningness-Eveningness-Stability Scale (improved) tries to do justice to all those requirements. It is a self-assessment instrument that determines the individual circadian preference and its amplitude on three continuous dimensions: Morning affect (MA), Eveningness (EV) and Stability/Distinctness (DI). The two dimensions MA and EV determine circadian preference, whereas the third dimension DI deals with the fluctuations during the day. Measuring M/E this way allows to account for the fact that M/E is a two-dimensional trait with an additional measure, the amplitude of the circadian preference through the day. The MESSi does not categorize participants into distinct groups; rather, it allows to consider the fact that a certain type of circadian preference may be more or less pronounced within an individual. In addition, it reflects that individuals may show fluctuations in their circadian preference depending on other, contextual factors. Therefore, the MESSi represents an innovative and potentially more valid way to determine circadian preference. For more and detailed information, see [16,20].

Despite its potential advantages, the MESSi requires further validation. One common way to validate self-assessments on circadian preference is to establish their relations with more objective measures of circadian typology, namely, physiological markers. The physiological markers that show strong daytime fluctuations and are hence sensitive to circadian rhythms are body temperature and the two hormones cortisol and melatonin. In the present paper, the relation between the MESSi and body temperature and cortisol level were established to further validate the instrument.

### 1.2 Circadian typology and body temperature

Body temperature has a very stable daily rhythm [21]. Therefore, we decided to use the body temperature as one of the physiological markers in this study.

In general, the body temperature is controlled by a complex feedback system [22]. The thermoregulatory system keeps the body temperature in balance between heat gain and heat loss [23]. The circadian regulation of the sleep-wake cycle is clearly associated with thermoregulatory mechanisms, but the circadian rhythm of the body temperature seems to be independent of the sleep-wake system [24].

To measure the rhythm of the body temperature, core body temperature, oral and skin temperature can be used. Usually, core body temperature is used, because its fluctuations are robust and less influenced by environmental issues. To measure the core body temperature, rectal measurements are often used. The participants have to wear small sensors [25,26] over a long period or even to swallow small data-logger pills [27]. These methods are unpleasant for the participants and rather expensive. Furthermore, frequent controls and surveillance of the participants are necessary, so they normally have to stay in a sleep lab. Alternatively, to have a more unobtrusive way of measuring and to determine the circadian rhythm of the body temperature under natural everyday condition, the skin temperature can also be assessed using small sensors. iButtons are often used for this purpose [28]. These are temperature data loggers that can be attached to a random location on the skin of the participants. iButtons measure the skin temperature within a range of – 40 to +85°C with a possible deviation of 0.5°C. The rhythm of the skin temperature can be influenced by environmental characteristics such as physical activity or environmental temperature. Above all, the acrophase seems to be quite robust [29]. The skin temperature fluctuations during a 24-h period normally show a wide range (between 31°C and 36°C) with the highest values appearing during sleep and the lowest during wake time. This can depend on the technique deployed by different sensors [29,30]. The skin temperature drops rapidly after awakening and decreases over the remainder of the day until reaching its minimum in the evening. During the night the skin temperature increases again and peaks in the early morning hours [29,30]. The aforementioned development of the body temperature across the day/night cycle describes the pattern that can be observed when averaging across people. However, there are individual differences in these shifts of the body temperature, some of which can be related to circadian preference. In particular, research has shown that M-types have earlier acrophase times (6 to 7 pm) in body temperature and their temperature minimum occurs also earlier (3 to 5 am) compared to E-types (acrophase: 8 to 10 pm, nadir: 5 to 6 am) [30,31,32,33,34]. It is yet an open question, whether measures of skin temperature reveal the same relations to circadian preference, given that they may be less sensitive than measures of core body temperature.

### 1.3 Circadian typology and cortisol

Another physiological marker that is known as a good indicator and whose relation to circadian preference is well established is the stress hormone cortisol. Cortisol is produced via the hypothalamus-pituitary-adrenal axis («HPA») and released into the bloodstream when the body needs energy [35,36]. Factors like anxiety [37] and light exposure [38] can strongly influence the secretion. Under normal conditions, the cortisol fluctuation follows a distinct rhythm with small (short duration) but strong (large amplitudes) episodes [39]. The cortisol level starts to increase in the second half of the night and peaks in the early hours of the morning. After that, it decreases slowly throughout the rest of the day and shows its nadir in the first half of the night [40]. This sharp increase in cortisol in the morning occurs immediately after awakening and reaches its highest level at least 30 minutes after awakening. This effect is called cortisol-awakening-response (CAR) [41]. Research is ambiguous about a potential influence of age and gender on CAR. It is assumed that the characteristics of the sample as well as the assessment methods influence the findings [39]. However, an influence of circadian typology on the cortisol level in the morning has been well established. In particular, M-types have a higher cortisol level after awakening than E-types [42,43,44]. This can be seen especially 30 and 45 minutes after awakening [45]. In addition, E-types feel less aroused than M-types at the same time in the morning [42]. Therefore, it is not surprising that the peak of cortisol level occurs earlier in M-types [8]. In addition, there is initial evidence pointing towards a difference in CAR between weekdays and weekend. Kunz-Ebrecht and her colleagues [46] showed that CAR is larger on working days compared to weekend days. They assume that the anticipation of a working day increases the CAR. In addition, the rise of the cortisol level after awakening is steeper on weekdays compared to days on the weekend [47].

### 1.4 Aims of the present study

The first and main aim of the present study was to further validate the measurement MESSi with the help of the physiological markers body temperature (in this case, skin temperature) and cortisol (here: cortisol levels in the morning and CAR). Randler and colleagues [16] proposed that it is important to validate the MESSi with more objective variables, such as actigraphy [48], body temperature and cortisol. Against this backdrop, we expect significant correlations between the sum-scores of the three dimensions of the MESSi with the nadir and acrophase of the skin temperature, as well as with the cortisol levels in the morning and the CAR.

Second, we aimed at determining the relation among circadian typology and the skin temperature, the cortisol levels in the morning and the CAR. We hypothesize a significant effect of circadian typology on the acrophase and nadir on the skin temperature, as well as on the cortisol levels in the morning and the CAR. M-types should present higher cortisol levels in the morning. The CAR should show a significant difference between the chronotypes, with Morning types having a greater CAR [8,30,31,32,33,34,42,43,44]. To investigate relations to chronotype, the reduced Morningness-Eveningness Questionnaire (rMEQ) [49] was used in addition to the MESSi, because, as mentioned before in the section 1.1, the MESSi’s conceptualization does not rely on a categorization of people according to a fixed typology. The convergent validity of the MESSi and the rMEQ could already be shown in an earlier study [48].

Third, we wanted to examine a potential effect of circadian typology and preference on the cortisol levels in the morning and the CAR between weekday and weekend. To this end, we assumed that there would be significant differences in the CAR between weekday and weekend moderated by participant’s chronotype. Fourth, we aimed at extending previous findings regarding differences in the CAR between weekday and weekend [46]. The cortisol levels in the morning and the CAR should show higher values on weekdays compared to weekend days.

The study is new and original in a way that it for the first time measures the relationship between these physiological variables and a three-dimensional measure of circadian preference. Usually, all studies in this respect are based on uni-dimensional measures that consider morningness-eveningness as one-dimensional. Here, we extend the findings based on three dimensions.

## 2. Material and Methods

### 2.1 Participants

A total of 97 university students (24 male and 73 female), aged 18 to 54 years, participated in the study. The mean age of the participants was 24.5 ± 6.0 years. We screened the whole sample by using the reduced Morningness-Eveningness Questionnaire (rMEQ) and the Morningness-Eveningness-Stability Scale improved (MESSi) to select a subsample of definite M and E-types (*N* = 42; mean age: 24.8 ± 5.8 years). Twenty-two M-types (5 male and 17 female) and twenty E-types (8 male and 12 female) could be identified. The majority of the males (84.6%) did not take any medication but 8 females indicated that they took hormonal contraception. The participants did not do any night or shiftwork and were excluded when they reached scores higher than 10 in the Pittsburgh-Sleep-Quality Index (PSQI). Twelve participants indicated to have a side job. One participant was excluded from the skin temperature analysis because he lost his device.

### 2.2 Questionnaires

#### 2.2.1 Morningness-Eveningness-Stability Scale, improved (MESSi) [16]

To determine circadian typology and its amplitude, the German version of the MESSi [16] was used. The MESSi contains three scales: Morning affect sub-scale (MA), Eveningness sub-scale (EV) and Distinctness/Stability sub-scale (DI). Each of those sub-scales is represented by 5 items with options ranging from 1 to 5. Higher scores represent higher expressions in the respective sub-scale. The MA sub-scale measures the affective facet of morningness-eveningness (“How easy is it for you to get up in the morning?”) and the EV sub-scale determines the affective facet of the evening orientation (“After waking up, I feel sleepy for some time”). The DI sub-scale gives information about the stability of the orientation of the participants. It shows how much the expression of the facets fluctuates throughout the day (“I can be concentrated at any time of the day”). Higher scores represent a greater fluctuation. Analysis showed a Cronbach’s alpha of 0.95 for the MA component of the MESSi, 0.90 for EV and 0.72 for the DI component. The MESSi has been used in some countries already, and the three-factorial structure has been established by psychometric analysis [20].

#### 2.2.2 Reduced Morningness-Eveningness Questionnaire (rMEQ) [49]

To determine circadian typology the shortened German version of the Morningness-Eveningness Questionnaire was used [50]. The questionnaire has 5 items. For the first question the participants has to mark a timespan, when they would like to get up (“If it was only for your own well-being and you could organize your day completely free, when would you get up?). The second question refers to the fatigue of the participants after getting up (“How tired do you feel in the morning in the first 30 minutes after awaking?”). Here they have to choose one of the four options ranging from “very tired” to “very awake”. For the third and the fourth question, the participants have to mark again a timespan concerning their need to sleep (“At what time do you get tired at night and feel the need to go to sleep?) and their feeling through the day (“At what time do you feel best?). With the last question, they give a self-assessment regarding their own chronotype. Options range from “clearly a Morning type” to “clearly an Evening type”. The participants can reach credit scores, ranging from 4 to 25. Classifications into one of the three chronotype categories are possible: morning types (18–25), neither types (12–17) or evening types (4–11). Analysis showed a Cronbach’s *α* of 0.84 for the sum-score of the rMEQ. In a further done study, the rMEQ was set in relation to the Composite Scale of Morningness to show convergent validity (*r* = 0.885) [51]. In the present study, the rMEQ was used to classify the participants into the different chronotypes groups.

#### 2.2.3 Pittsburgh-Sleep-Quality Index (PSQI) [52]

To determine the individual sleep quality of the participants, this 4-week-retrospective questionnaire was used. The 19 items require a subjective rating of seven sleep-related categories: subjective sleep quality, sleep latency, sleep duration, habitual sleep efficiency, sleep disturbances, use of sleeping medication, and daytime dysfunction (e.g. “How many times did you sleep badly because you woke up in the middle of the night or early in the morning?”). The 5 items related to peer/mate assessment were not included. The total scores can range from 0 to 21. Higher scores represent a worse sleep quality. The cut-off score for a bad sleep quality can be located at 5. A score higher than 10 suggests an impaired sleep disorder. Analysis of the study from Buysee et al. (test-retest reliability) showed high correlations of the global PSQI scores (*r* = 0.85). The PSQI represents a sensitivity of 89.6% and a specificity of 86.5% [52]. In the present study, the PSQI was only used to control for sleep disorders. No further calculations were done.

### 2.3 Physiological measures

#### 2.3.1 Skin temperature

The skin temperature of the participants was measured by iButtons (Thermochron Temperature Data Loggers, type DS1922L, Maxim Integrated Products, Munich; Germany) [53] to determine natural fluctuations because the devices can be used in the participants’ home environment. The iButtons were attached to the inside of the non-dominant wrist of the participants using one-size sweatbands (HEAD, Kennelbach, Austria) to reduce most of the environmental influences. The skin temperature was recorded for seven days (00.00 am to 23:59 pm) every 2 minutes. Using the corresponding software (1-Wire, version 03.19.47) the data were extracted from the device. For every participant there were 720 temperature logs per day (5,040 in total). For each 24-h section temperature data were averaged into 20-min sections and missing data were interpolated. Temperature logs showing less than 28 degrees Celsius and higher than 40.8 degrees Celsius were excluded. The participants received the instruction to wear the device constantly (except for showering or taking a bath) and to note the timespan when they had to remove it. The temperature logs were visually screened for those timespans and the corresponding data were excluded.

#### 2.3.2 Saliva cortisol

The cortisol levels of the participants were assessed in their saliva by using salivettes (Sarstedt, Nümbrecht, Germany). To this end, participants were asked to sample four saliva probes; two during the week and two on the weekend. The first probe on each day was to be taken immediately after awakening and the second one half an hour later. The participants were told not to eat, drink (other than water) or brush their teeth in these 30 minutes and not to exercise. They had to mark the label on the polypropylene container of the salivettes with the date and the number of the probe (first or second). The saliva probes were sent by post to a laboratory (Dresden Lab Service GmbH, Dresden, Germany) for analysis. They calculated the cortisol level in nanomol per liter for each participant of each probe and sent the results via mail.

### 2.4 Procedure

The study took place at the University of Tuebingen, Germany in the survey period from January 1^st^ to March 31^st^, 2017. The recruitment information was given to all students of the university via mail and posters at the campus. Interested students were invited for a first screening. The sessions began for the participants with their agreement for the conditions of participation and the assessment of their demographic variables (age, gender, relationship status, handedness, occupation and medication). After that, they completed the rMEQ, the MESSi and the PSQI to identify the individual chronotype (and its amplitude) and exclude participants with sleep disorders. If the participants met all our criteria (especially definite M or E-type, no shift work and no sleep disorder), a second appointment was arranged. At this appointment the iButton was attached and the salivettes were handed out (4 for each participant). Participants got detailed information how to handle the devices and not to forget to note when they remove the iButton. After one week of sampling, a third appointment was arranged at which the participants returned the devices.

### 2.5 Data analysis

Mean differences for the participants’ characteristics (age, gender, relationship status, PSQI and MESSi components) as a function of chronotype were determined by calculating t-statistics and chi-square statistics. To estimate temperature parameters (acrophase and nadir) a curve-fitting cosinor procedure was applied and summary measures of R^2^ (the variance statistic or goodness of fit of the actual data to the approximating 24h cosine curve) were calculated. The acrophase/nadir of the skin temperature represents the average acrophase/nadir of the skin temperature for each participant in the whole sampling time. Additionally, the amplitude (difference between acrophase and nadir) of the skin temperature was determined for each participant. Furthermore, Pearson correlations (zero-order) were calculated to account for the relationship of the dimensions of the MESSi with the skin temperature parameters and the cortisol parameters: CAR on a weekday, CAR on the weekend, first cortisol probe directly after awakening on a weekday (t1_weekday), second cortisol probe 30 minutes later on a weekday (t2_weekday), first cortisol probe directly after awakening on the weekend (t1_weekend) and second cortisol probe 30 minutes later on the weekend (t2_weekend).

Multivariate variance analyses (MANOVA) were run to assess the effect of chronotype on the skin temperature and the cortisol parameters. Chronotype (M-types vs. E-types) represents the independent variable and the skin temperature parameters (acrophase, nadir, amplitude) as well as the cortisol parameters the dependent variables. Partial eta-squared was used as a measure of effect size. All statistical analyses were conducted using SPSS version 25 (IBM Corp., Armonk, New York; released 2017).

## 3. Results

### 3.1 Participant characteristics

To give further information, the participants’ characteristics are listed in Table 1. It shows the characteristics of the participants split by Chronotype (The classification of the participants into their chronotypes is based on the values of the rMEQ). M-types scored significantly higher for the MA component of the MESSi. E-types showed significant higher values for the EV component of the MESSi. No significant group differences were found for age, gender, relationship status and the DI component of the MESSi.

**Table 1:** Descriptive statistics (*M, SD, n* and %) and test statistics for participant characteristics as a function of chronotype.

	M-types	E-types	Test statistic

Age (in years)	*M* = 24.86 (7.07)	*M* = 24.65 (4.25)	t_(40)_ = .12, *p* = .905
*Gender*			
Male	n = 5 (22.7%)	n = 8 (40.0%)	X^2^ = 1.46, *p* = .227
Female	n = 17 (77.3%)	n = 12 (60.0%)	
*Relationship status*			
Single	n = 11 (50.0%)	n = 9 (45.0%)	X^2^ = 0.49, *p* = .782
Relationship	n = 10 (45.5%)	n = 9 (45.0%)	
Married	n = 1 (4.5%)	n = 2 (10.0%)	
*Dimensions of the MESSi*			
MA	*M* = 21.64 (2.12)	*M* = 10.70 (2.45)	t_(40)_ = 15.44, *p* < .001
EV	*M* = 10.86 (3.37)	*M* = 19.85 (2.54)	t_(40)_ = –9.81, *p* < .001
DI	*M* = 18.27 (3.82)	*M* = 20.30 (3.08)	t_(40)_ = –1.90, *p* = .065

### 3.2 Validation of the MESSi

Correlation analysis showed no significant correlation between the rMEQ sum-score and the skin temperature parameters (acrophase: *r* = .03; nadir: *r* = .05 and amplitude: *r* = –.02). The MA and EV component of the MESSi also showed no significant correlation with the skin temperature parameters. The DI component, on the other hand, showed a weak negative correlation with the nadir of the skin temperature (*r* = –.39, *p* = .05), indicating that higher fluctuations in circadian preference are associated with a lower average skin temperature during nadir. DI showed no further significant correlation with the skin temperature parameters (acrophase: *r* = –.12 and amplitude: *r* = .24). Separated by Chronotype, DI was negatively correlated with the nadir of the skin temperature (*r* = –.50, *p* = .05) for E-types. No further significant correlations for the components of the MESSi and the skin temperature parameters were found, even when split by Chronotype.

Correlation analysis showed no significant correlation between either the sum-scores of the rMEQ with the cortisol levels in the morning (first cortisol probe directly after awakening and second cortisol probe 30 minutes after awakening) and CAR or the components of the MESSi with those variables.

### 3.3 Relation of Chronotype and physiological parameters

In Table 2 the descriptive statistics of the skin temperature and its nadir/acrophase, as well as the amplitude is shown split by Chronotype. There is a significant difference in the average skin temperature. M-types showed a higher overall skin temperature than E-types.

**Table 2:** Descriptive and test statistic (*M* and *SD*) of the temperature (mean value in Celsius), nadir and acrophase values of the temperature (in Celsius) and the amplitude of the temperature.

	M-types	E-types	Test statistic

Overall temperature	*M* = 33.41 (1.96)	*M* = 33.29 (1.71)	t_(19624)_ = 4.53, *p* < .001
Temperature during nadir	*M* = 32.22 (1.08)	*M* = 32.13 (0.99)	t_(39)_ = 0.27, *p* = .787
Temperature during acrophase	*M* = 34.57 (0.90)	*M* = 34.40 (0.83)	t_(39)_ = 0.64, *p* = .527
Amplitude	2.36 (1.40)	2.27 (1.02)	t_(39)_ = 0.23, *p* = .828

**Figure 1:**
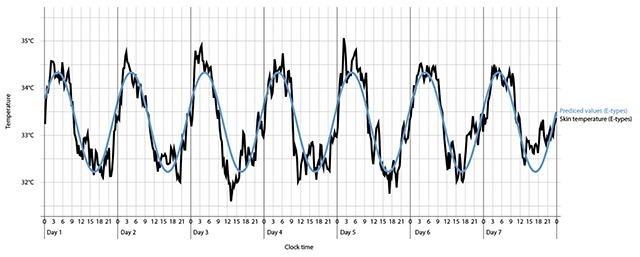
Actual and predicted skin temperature values for E-types.

**Figure 2:**
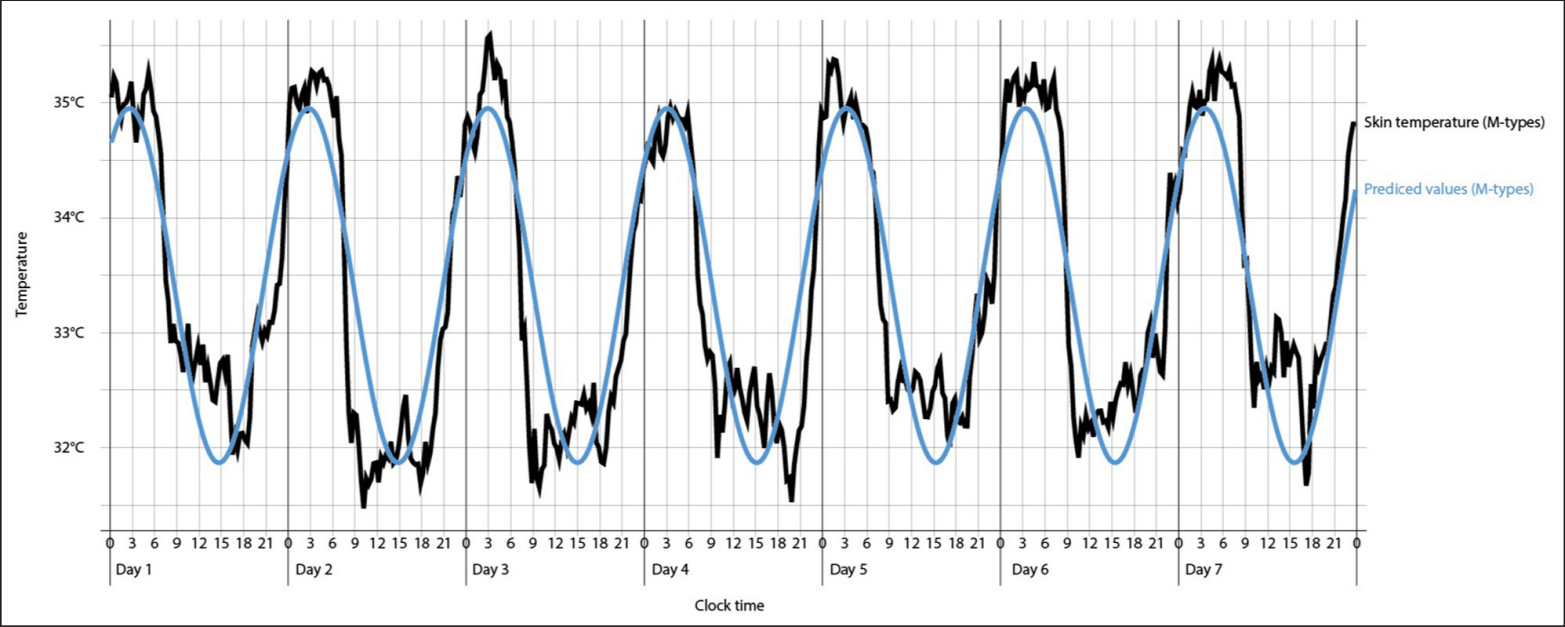
Actual and predicted skin temperature values for M-types.

The cosinor analyses of the skin temperature values showed a good fit (R^2^ = .24) of the actual temperature data to the approximating 24 h cosine curve. In separate analyses by Chronotype, M-types showed a better fit (R^2^ = .31) than E-types (R^2^ = .19), see Figures 1 and 2.

The MANOVA showed no significant effect of Chronotype on the cortisol levels in the morning (first cortisol probe directly after awakening and second cortisol probe 30 minutes after awakening) and the CAR. For detailed information, see Table 3.

**Table 3:** Test statistic of the MANOVA (df, *F, p*-values and effect size) for the effect of Chronotype on the cortisol levels in the morning and the CAR.

Variables	df	*F*	*p*	η_p_^2^

t1	1, 38	2.99	.092	.073
t2	1, 38	0.05	.833	.001
CAR	1, 38	0.94	.340	.024

t1 = first cortisol probe directly after awakening; t2 = second cortisol probe 30 minutes after awakening; CAR = Cortisol-Awakening-Response.

### 3.4 Relation of Chronotype and circadian preference among cortisol levels in the morning and CAR depending on weekday

None of the components of the MESSi (MA, EV and DI) showed significant correlations with the cortisol levels in the morning (first cortisol probe directly after awakening and second cortisol probe 30 minutes after awakening), neither on the weekday nor on the weekend. The CAR on a weekday and on the weekend showed also no significant correlations with the components of the MESSi (MA, EV and DI).

In separate analyses by Chronotype, M-types showed a positive correlation of EV (*r* = .48, *p* = .05) with the first cortisol probe directly after awakening on a weekday. E-types showed a positive correlation of EV (*r* = .62, *p* = .01) with the first cortisol probe directly after awakening on the weekend. The CAR on the weekend showed a negative correlation with EV (*r* = –.54, *p* = .05) only for E-types.

Chronotype showed a significant effect on the first cortisol probe directly after awakening on a weekday (*F_1_*_, 35_ = 6.38, *p* = .016, *η_p_^2^* = 0.14). M-types (*M* = 17.44, *SD* = 7.28) presented significant higher cortisol values after awakening than E-types (*M* = 12.18, *SD* = 5.53). Further analysis showed no significant effect of Chronotype on the other cortisol levels in the morning (second cortisol probe 30 minutes after awakening on a weekday: *F_1_*_, 38_ = 0.004, *p* = .952, *η_p_^2^* = 0.00; first cortisol probe directly after awakening on the weekend: *F_1_*_, 38_ = 0.52, *p* = .475, *η_p_^2^* = 0.014; second cortisol probe 30 minutes after awakening on the weekend: *F_1_*_, 38_ = 0.21, *p* = .646, *η_p_^2^* = 0.006). Chronotype showed also no significant effect on the CAR, neither on a weekday (*F_1_*_, 38_ = 1.87, *p* = .197, *η_p_^2^* = 0.05) nor on the weekend (*F_1_*_, 38_ = 0.04, *p* = .847, *η_p_^2^* = 0.001).

### 3.5 Cortisol and weekday

Analysis showed a significant difference between the second cortisol probe 30 minutes after awakening on a weekday and the second cortisol probe 30 minutes later on the weekend (t_39_ = 4.45, *p* < .001, *d* = .384). The cortisol level 30 minutes after awakening on a day during week was significantly higher (*M* = 28.11, *SD* = 12.22) than the cortisol level 30 minutes after awakening on the weekend (*M* = 19.26, *SD* = 10.22). There were no significant differences between the first cortisol probes directly after awakening concerning weekday (t_39_ = –0.19, *p* = .847, *d* = .365).

The CAR showed a significant difference between the cortisol levels on a day during week and the weekend (t_39_ = 3.91, *p* < .001, *d* = .312). The CAR on a weekday (*M* = 13.04, *SD* = 12.80) reached higher cortisol levels than the CAR on the weekend (*M* = 3.89, *SD* = 12.39).

## 4. Discussion

### 4.1 Validation of the MESSi

Regarding the main aim of the present study (to further validate the MESSi), previous results [48,54] could be significantly extended. In an earlier study, high correlations between the MA and the EV component of the MESSi with the scores of the rMEQ were found (for more detailed information, see [48]). In this study, morning-oriented participants showed significant higher scores for the MA component of the MESSi and evening-oriented participants for EV component, which confirms the validity of the MESSi. The DI component showed no significant difference between morning and evening-oriented participants. Nevertheless, with a *p*-value of *p* = 0.065 a trend can be seen. This trend suggests that evening-oriented participants show higher fluctuations in their daily circadian preferences. So far, there is no exact explanation for this finding and therefore requires further research. However, it is suspected that morning-oriented people can better integrate their behavior into our “social clock driven society” [55, pp. 11]. Our daily life is determined by time schedules (in school, at university or at work) and evening-oriented persons are forced to adapt their behavior to those. For this reason, larger fluctuations in the behavior of circadian preference could be seen. To further validate the MESSi, correlations between the sum-score of the rMEQ/the components of the MESSi and the skin temperature parameters were calculated. The rMEQ sum-score as well as the MA and EV component of the MESSi showed no significant correlations. At this point, it should be mentioned that only extreme chronotypes (M and E-Types) were considered for analysis. There might be stronger correlations if all chronotypes were considered (Morning types, Evening types and Neither types). Further research could take this into account.

The DI component of the MESSi, on the other hand, showed a weak negative correlation with the nadir of the skin temperature. This suggest that higher fluctuations in circadian preference might be associated with a lower skin temperature during nadir. A further interesting finding was the negative correlation of the DI component with the nadir of the skin temperature in E-types. Evening-oriented participants showed a lower temperature in the nadir phase, when their fluctuations in their circadian preferences are larger. The stability of the circadian phase during the day for evening-oriented participants could be associated with a physiological marker. This is a new finding so far and needs further consideration. In addition, it gives an interesting insight into the skin temperature regulation, probably moderated by circadian preference. Evening-oriented people tend to show greater fluctuation in their circadian behavior throughout the day. These larger fluctuations could affect the rhythm of the skin temperature. Thus, the greater efforts that evening-oriented people have to make in order to keep up in everyday life may affect their skin temperature rhythm. This greater effort can lead to stress and sleep deprivation in evening-oriented people and these factors can affect the rhythm of the skin temperature [58,59,60].

### 4.2 Relation of Chronotype and physiological parameters

M-types showed a better fit in their actual temperature to the approximating data than E-types. This suggests that M-types have a more “regular rhythm” of skin temperature with smaller fluctuations. This could be due to a lower amplitude. It could be shown, that E-types tend to have a lager amplitude compared to M-types [9]. In this study, no significant difference between chronotype and amplitude could be seen. In general, M-types showed a significant higher temperature than E-types. Even Horne and Östberg in 1976 [12] could show that M-types tend to have a higher daytime temperature than E-types. One might argue that this result is caused by the female-based sample with more women in the morning type group and more men in the evening type group. It could be shown that women tend to have a higher average body temperature in a 24-h rhythm compared to men and the circadian rhythm of the body temperature is influenced by the menstrual phase of females [56]. However, it should be considered that the skin temperature is less influenced by the female menstrual cycle compared to the core body temperature [57]. Important for further analysis would be a balanced sample controlling for the use of contraceptives and the menstrual cycle.

The results of the calculated MANOVA showed no effect of circadian typology on the cortisol levels in the morning and the CAR at all. One possible explanation for these findings might be the fact that only university students participated in the present study. In a study of Randler and Schaal [44] it could be found out that bed times are one of the most relevant variables that affect cortisol levels. Adolescents had to get up earlier and therefore sample the probes for cortisol determination at earlier times in the morning. Generally higher cortisol levels for adolescents are the result. Another important factor is the circumstance that in the present study the cortisol sampling was not controlled for a special weekday. As it can be read in the review of Clow and colleagues [61], a stressful weekday can influence the cortisol levels in the morning. Further studies should consider this fact and let the participants sample their probes on given days.

### 4.3 Relation of Chronotype and circadian preference among cortisol levels in the morning and CAR depending on weekday

None of the components of the MESSi, neither on the weekday nor on the weekend, showed significant correlations with the cortisol levels in the morning and the CAR. In the present study, cortisol was measured twice on two single days: on one day during week and on one day on the weekend. Further research should consider to measure cortisol on seven constant days.

In order to be able to view the results in more detail, the scoring on the components of the MESSi (MA, EV and DI) was presented separately by Chronotype. These findings were then connected with the values of the cortisol levels in the morning. M-types that scored high on the EV component also have higher values in cortisol directly after awakening during a weekday compared to M-types that scored low on EV. This suggests that a higher “evening” orientation (represented by higher scorings on the EV component of the MESSi) must be compensated with higher cortisol values after awakening to be better activated.

Interestingly, E-types that scored high on EV showed a positive correlation with the first cortisol sample directly after awakening on the weekend. Consequently, a negative correlation of EV with the CAR on the weekend for E-types was found. The cortisol values directly after awakening on the weekend might be high enough for an optimal activation. One possible explanation could be that on the weekend E-types can get up at a time that better corresponds to their inner rhythm and therefore a “good” activation by cortisol can be observed [44]. On the other hand, the circadian rhythm of the M-types is better adapted to the demands of everyday life [62]; hence, they do not have such a vast conversion of their circadian biological rhythm from weekday to weekend compared to E-types.

In line with Randler and Schaal [44], a significant influence of Chronotype was present for the first cortisol values directly after awakening on a weekday. M-types showed significant higher cortisol values than E-types. This could be one possible explanation, why M-types get up easier in the morning [3]. From the beginning, their first cortisol values are higher as compared to E-types, so their body can be better activated. In contrast, E-types are forced to get up to earlier for their inner rhythm and thus their natural activation by an increased cortisol level might fail (Randler und Schaal [44]).

### 4.4 Cortisol and weekday

The analysis of the difference between the cortisol values on a weekday and on the weekend independent of Chronotype and circadian preference showed a significant difference between the cortisol values 30 minutes after awakening. The values on a weekday were significantly higher than the corresponding values on the weekend. This effect can also be observed with the CAR. In line with other studies [47,63] the CAR on a weekday reached significantly higher cortisol levels than the CAR on the weekend. This means that during the week a higher activation of the body is necessary to start into the day. Some researchers attribute this difference to factors such as stress or anxiety at work [61,64]. Another possible explanation could be due to the fact that the participants wake up at later times at weekends, as they usually do during the week. However, their body gets used to the wake-up times during the week and activates the body at the usual time with an increase of the cortisol level. If then the cortisol level is measured at a later time, the usual increase may have fallen again.

### 4.5 Limitations

In the present study, chronotype was assessed by self-reported data with two questionnaires (rMEQ and MESSi). Future work can focus on using actual sleep data from objective devices, such as actigraphy [33]. Further, we analyzed the skin temperature rhythm for the present study. One could consider determining the core body temperature because it is less affected by disturbing variables [22,23,24]. It could also be considered to collect information about the state of the participants and therefore control for stress or actual health [46]. The saliva cortisol probes were collected on one day during the week and on one day on the weekend. It would be more prudent and recommendable to sample the cortisol levels every day for one week to be able to determine more external influences. Finally, it should be noted that further studies should pay attention to a gender-balanced sample.

### 4.6 Conclusion

The present findings demonstrate that the skin temperature is weakly associated with morningness-eveningness, and the stability of the circadian phase might have a potential effect on the skin temperature rhythm. It could be shown that physiological markers, such as cortisol levels, are intertwined in our circadian phase and that other factors (weekday vs. weekend) might play an important role for the activation in the morning. However, it should also be noted that the primary aim of the present study, namely to further validate the MESSi with physiological parameters like skin temperature and cortisol levels, was not sufficiently satisfactory in this sample. Further analyses and investigations are planned to do justice to this project.
